# High growth temperature for AlN by jet stream gas flow metalorganic vapor phase epitaxy

**DOI:** 10.1038/s41598-023-29150-6

**Published:** 2023-02-10

**Authors:** Kentaro Nagamatsu, Takumi Miyagawa, Atsushi Tomita, Hideki Hirayama, Yuusuke Takashima, Yoshiki Naoi

**Affiliations:** 1 Institute of Post-LED Photonics, Tokushima University, 2-1 Minami-Josanjima, Tokushima, 770-8506 Japan; 2 Graduate School of Advanced Technology and Science, Tokushima University, 2-1 Minami-Josanjima, Tokushima, 770-8506 Japan; 3 Institute of Physical and Chemical Research, RIKEN, Wako, 351-0198 Japan

**Keywords:** Engineering, Electrical and electronic engineering

## Abstract

Deep ultraviolet light-emitting diodes have attracted considerable attention for realizing virus inactivation applications. The UV-LEDs use the AlN underlying layer and the plane sapphire substrate. However, the low growth temperature in AlN underlying layer is grown by limited growth temperature in conventional MOVPE, and high temperature is preferable for AlN growth. Furthermore, the AlN underlying layer has many dislocations owing to the active layer in the device region when the flat sapphire substrate was used with a dislocation value of > 10^9^ cm^−2^. We showed the high-temperature crystal growth of AlN with a temperature of 1700 °C by high temperature and gas flow velocity MOVPE. The achieved dislocation density was ~ 4 × 10^8^ cm^−2^. Additionally, this data means the low dislocation densities in the AlN layer with a growth time of only 15 min and a dislocation density of < 1 × 10^9^ cm^−2^ are obtained. The AlN growth temperature exceeding 1550 °C decreases the growth rate. These results indicate desorption from the surface of the substrate in a hydrogen atmosphere. Furthermore, the characteristic dislocation behavior of AlN in high-temperature growth at 1700 °C was elucidated from TEM images.

## Introduction

AlGaN-based ultraviolet LEDs in the UVC region have attracted interest for viral inactivation and sterilizing applications, which are expected to be the largest market in UV-LEDs^1,2^. Recently, numerous groups found an inactivation impact for SARS-CoV-2; the LEDs are anticipated to increase demand^3–5^. However, the cooling systems are vital for heat generation from LEDs because the LED efficiency in the UVC region is very low compared with that of the visible region^2,6–8^. Therefore, the major advantages of LEDs’ compact size have been compromised. Furthermore, nonradiative recombination, which is impacted by dislocation, is a cause of heat generation^9,10^. Most dislocations are through the active layer in devices without generating loops for each other due to incomplete growth conditions in the AlN underlying layer^11,12^. Therefore, many institutes have proposed several approaches to enhance the crystalline quality of AlN underlying layers, including migration enhancement growth, ammonia pulse growth, and low-ammonia supply growth^13–17^. The migration enhancement growth is efficient for minimizing parasitic reactions; however, a high growth rate is challenging in principles^15,18,19^. Nevertheless, ammonia pulse growth and low-ammonia supply growth have a trade-off between parasitic reactions and growth temperature, which is influenced by the growth conditions selected^11,14,16^. Additionally, the previously found AlN growth is low growth temperature for AlN material, and it is not verified as reducing the growth rate by typical desorption as observed in other materials^20^. Meanwhile, Miyake et al. reported low dislocation AlN growth by the sputter-anneal method, which method realized high-temperature with the temperature of 1700 °C by separating in AlN deposition and crystallization^21,22^. However, this approach cannot be continuously grown in the device structure and requires renewal by eliminating it from the chamber. Recently, our group developed a growth approach with a low-parasitic reaction at a temperature of 1500 °C by metal–organic vapor phase epitaxy (MOVPE) imitating jet gas stream^23^.

This study evaluated high-temperature growth in AlN by Jet stream gas flow MOVPE. Furthermore, the dislocation behavior of the grown AlN was analyzed by Transmission Electron Microscope (TEM).

## Results and discussion

### The realization of high temperature for AlN growth by MOVPE

**Figure 1:**
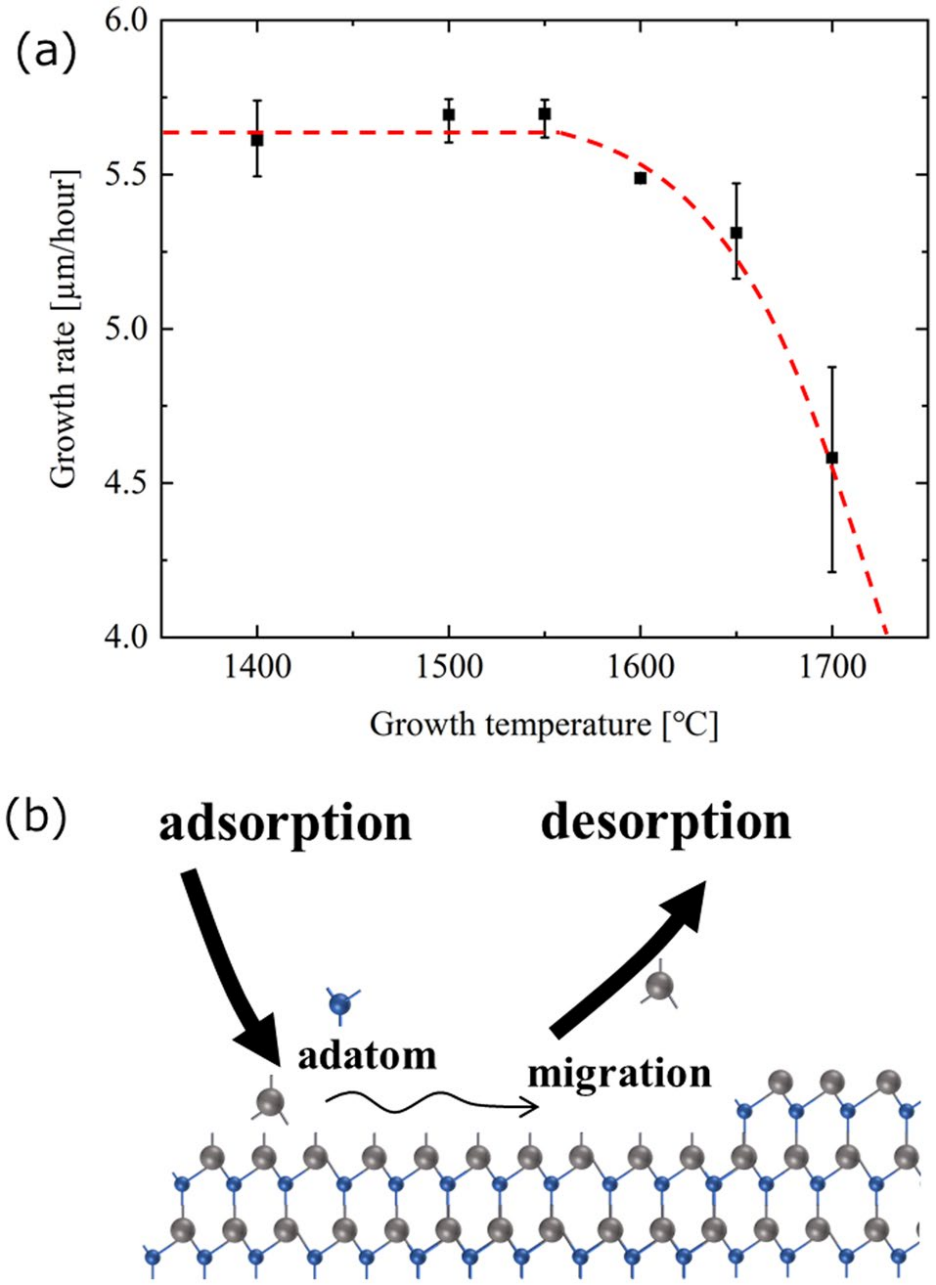
The dependences on growth temperature for the growth rate. (**a**) The average AlN growth rate with several growth temperatures at in-plane 5-point data. The error bars in each data are maximum and minimum data. The data of 1700 °C is obtained from the 3 points without 0 (center) and 5 mm points. (**b**) The schematic image of crystal growth in the vicinity of the surface.

**Figure 2:**
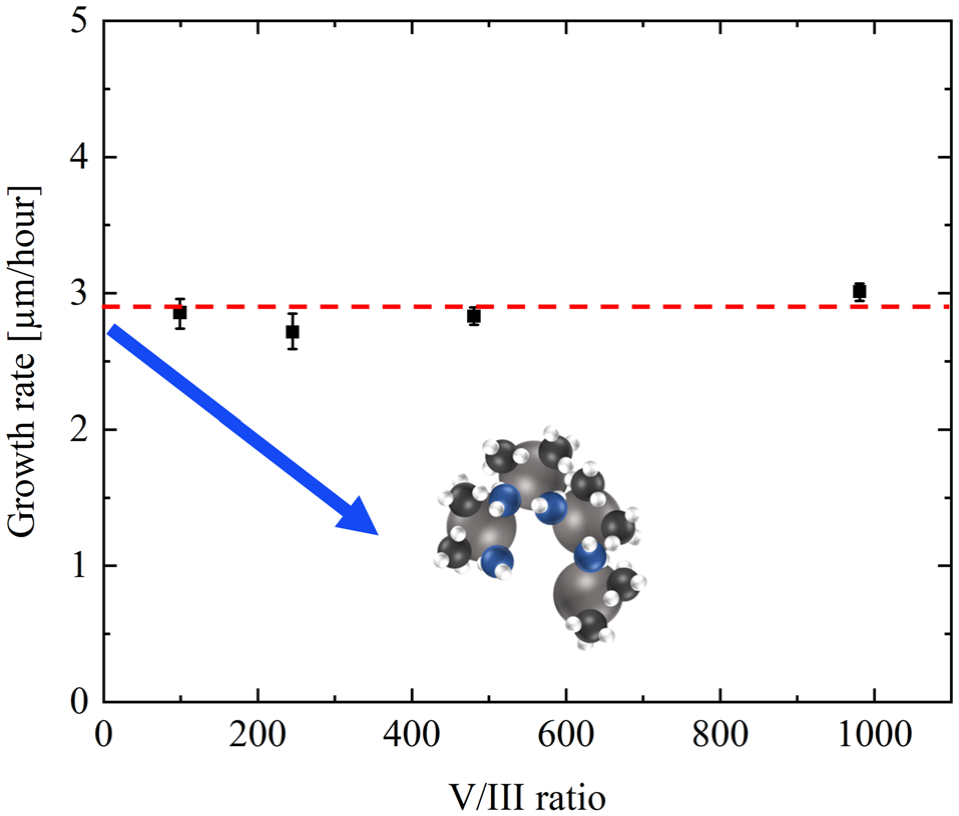
The AlN growth rate as the function of the V/III ratio: The growth rate decreases when the parasitic reaction in the vapor phase, including in the blue arrow. These data indicate the AlN growth at 1700 °C is unaffected by the parasitic response in this experiment.

To realize high temperatures for AlN growth on the c-plane sapphire substrate, we used Jet stream gas flow MOVPE^23^. Firstly, optimized growth temperature in AlN was explored. AlN was grown at different growth temperatures from 1500 to 1700 °C following the grown AlN buffer layer. Trimethyl aluminum (TMA) and ammonia were used as the precursors. The TMA flow rate was 209 μmol/min, and the V/III ratio is 213 during the AlN layer growth. The AlN growth in 1400 °C is used 2-step growth after obtaining a step flow surface with the growth temperature of 1500 °C for surface morphology. Figure 1(a) shows the AlN growth rate as a function of growth temperature. From 1550 °C, the growth rate decreased with increasing temperature despite nearly a similar growth rate with less than 1550 °C. This finding can be elucidated by two phenomena shown below. Rising temperatures cause a parasitic reaction in the vapor phase, which is a phenomena^23–27^. The TMA raw material may accelerate the reaction with ammonia during transit by increasing the growth temperature^28^. Subsequently, these reactions are not contributed to an AlN growth in case of the formation of polymerization^23,24^. Moreover, the other one is the desorption of the Al adatom. During crystal development, the adatom undergoes adsorption and migration is integrated at the atomic step layer in the nearby solid state^29^. Additionally, the adatom is deserted if the surface energy is higher than that (Fig. 1b)^30^. Generally, crystal growth is preferred closer to an equilibrium condition due to superior crystalline quality, albeit the growth rate becomes slightly slower. Therefore, the influence of the parasitic reaction on AlN growth at 1700 °C was evaluated. If the growth rate affects the parasitic response, the higher V/III ratio is more vulnerable to the parasitic effect^28,31^. Subsequently, the TMA supply decreased to 91 μmol/min, and the V/III ratio was widely investigated with several ammonia flow rates. Therefore, the growth rate is confirmed not to decrease as the V/III ratio from 98 to 980 (Fig. 2). These results show that the growth method is stable with the unaffected parasitic reaction at 1700 °C. Hence, the decreased AlN growth rate over 1600 °C is indicated by the desorption of adatom on the surface. Additionally, the desorption starting temperature result is almost the same value as the previous report by calculations^32,33^.

### The analyzed dislocation densities and behavior of dislocations in high temperature AlN growth

**Figure 3:**
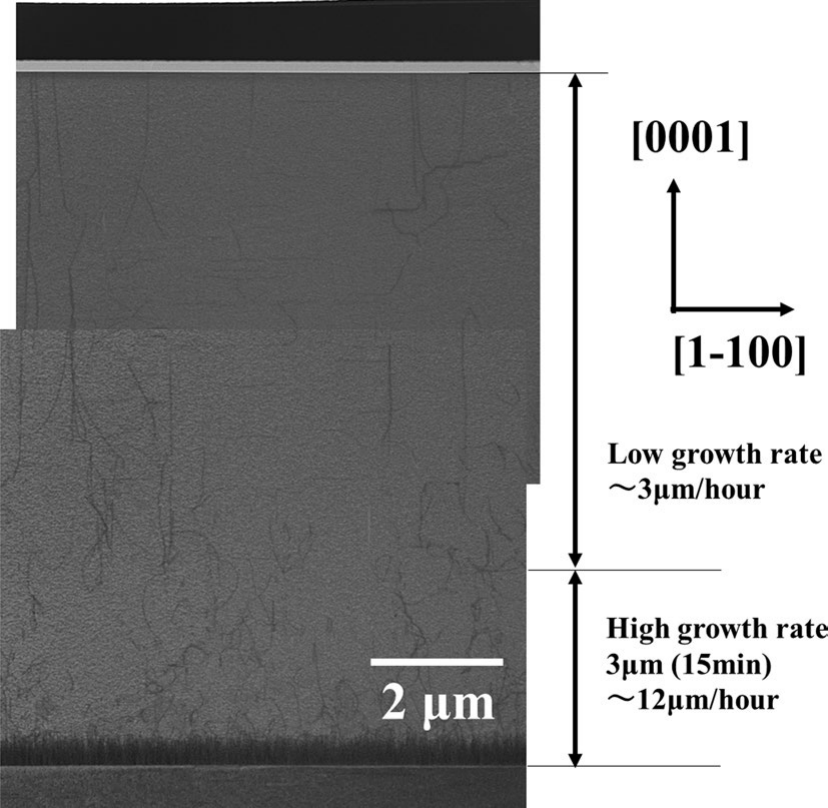
The cross-sectional TEM image from sapphire to AlN top surface at bright field overall. The depth and width sizes are 300 nm and 7.9 μm, respectively, in each picture. From the sapphire, the initial 3 μm and from that until the surface are different growth conditions at the high and low growth rates.

**Figure 4:**
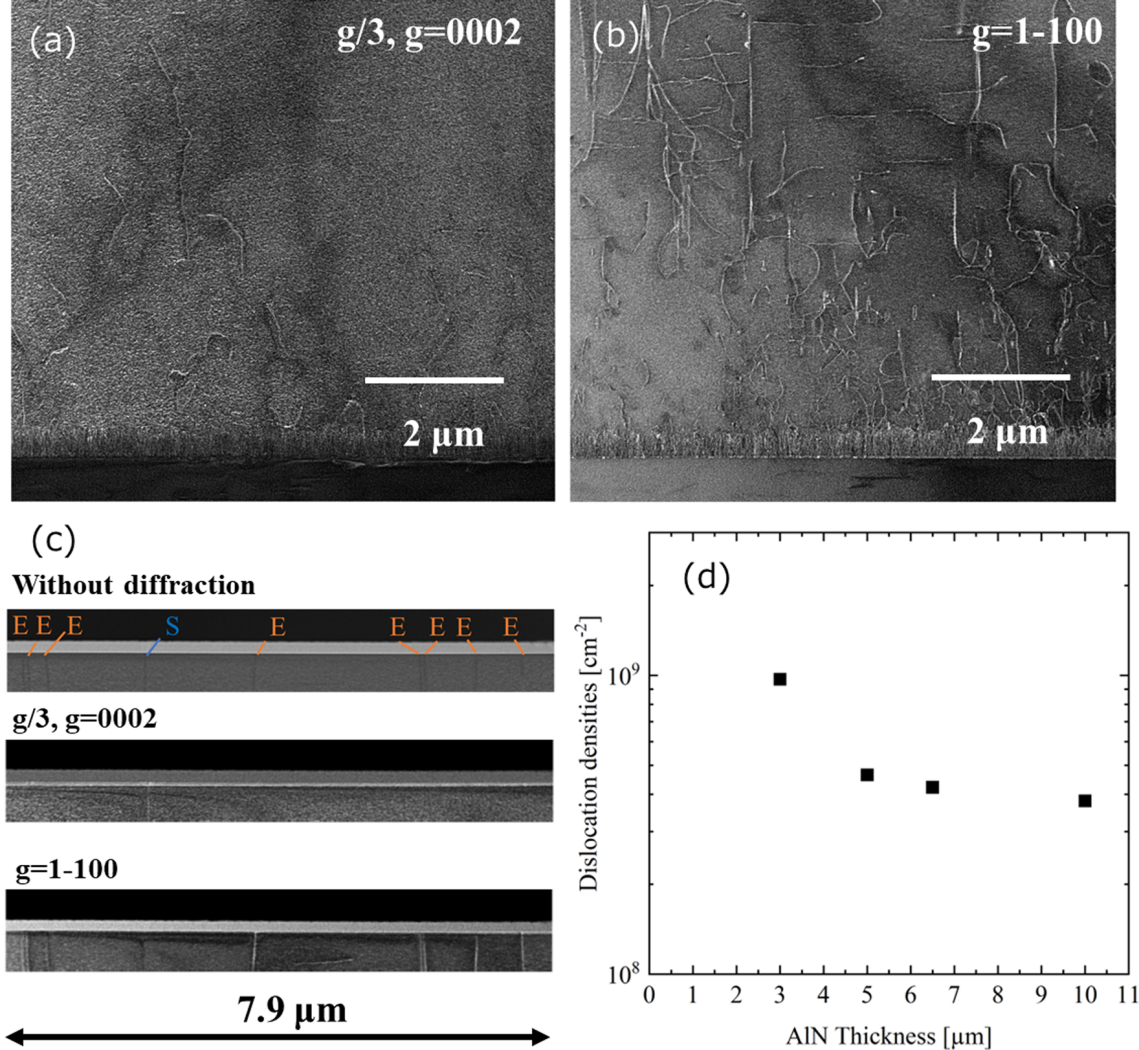
The measurement of the dislocation densities by cross-sectional TEM images under two-beam conditions at weak excitation. (**a**) The cross-sectional TEM image at the dark field with the g/3 and g = [0002] axis. (**b**) The cross-sectional TEM image at the dark field with the g = [1–100] axis. The TEM images in (**a**) and (**b**) are measured in the same regions. The measurement volume is 7.9 μm × 300 nm × 7.2 μm, respectively. (**c**) The analysis method in dislocation densities and the dislocation type. This TEM image is focused on the close to the surface region. (**d**) The total dislocation density including the screw, edge, and mixed depth profiles from the TEM images.

As shown in Figs. 1 and 2, the high temperature for the AlN is achieved by MOVPE at 1700 °C. Subsequently, the efficiency of reducing dislocation density is investigated with AlN growth at 1700 °C. The AlN buffer layer is grown at 300 °C, and the 1st AlN layer is grown at a temperature of 1700 °C for 15 min. The thickness of the buffer layer is assumed to be about 10 nm by the growth time from the same growth condition thick layer. The 2nd AlN layer is grown at 1700 °C for 150 min. The TMA flow rates of the 1st and 2nd AlN layers are 341 and 91 µmol/min, respectively. Figure 3 shows a cross-sectional TEM image at 1700 °C grown AlN on a *c*-plane sapphire substrate. The images are superimposed on two pictures to indicate the overall structure because the AlN thickness was approximately 10 μm. Several dislocations were clearly reduced at initial 300 nm. Moreover, the dislocations also seemed active to be crooked from 300 nm to 5 μm. Accordingly, the dislocation density is reduced by the rising temperature at 1700 °C. For detail discussion, dislocations were measured under two-beam conditions with g/3, g = [0002] (Fig. 4a), and g = [1–100] (Fig. 4b) by dark-field TEM images. These dislocations in the g vector axis [0002] are few compared with the g vector axis [1–100]. Additionally, both types of dislocation were found to be well flexed. Particularly, the dislocation of the upper side over 3 µm with the low growth rate region is verified to grow the m-axis.

Specifically, the dislocation is counted for each diffraction axis from the TEM image to observe dislocation densities, as shown in Fig. 4(c). This sample’s thickness at the depth direction is processed at 300 nm for measuring TEM images. The edge and screw type dislocations are 3.4 × 10^8^ and 4.2 × 10^7^ cm^−2^, respectively. From the result, the total threading dislocation density in the top surface of AlN is 3.8 × 10^8^ cm^−2^. Due to understanding dislocation information as the depth axis, the dislocation densities were measured at several thicknesses of 3, 5, and 6.5 μm in Fig. 4(d) at the same as Fig. 4(c). The dislocation density decreased as the growth thickness increased. The data of 10 μm is the top surface of AlN. From the finding, the dislocation density of growth time at only 15 min is less than 10^9^ cm^−2^ from the value of 3 μm. In addition, the full width at half maximum by XRC 0002 and 10–12 in this sample were 133 and 163 arcseconds, respectively. The XRC result is also indicated in the first half of 10^8^ cm^−2^.

**Figure 5:**
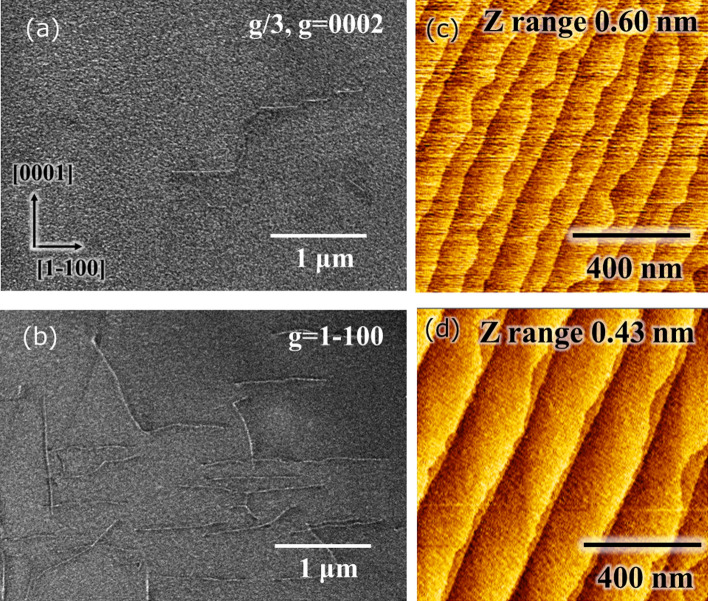
The TEM images focus on the dislocation in grow m-axis and the AFM images. (**a**) The cross-sectional TEM image of a dark field with the g/3, g = [0002] (**b**) The cross-sectional TEM image of a dark field with the g = [1–100] (**c**) AFM image in case of taking-off from the reactor after AlN growth at initial three μm by the high growth rate. The AFM image is a different sample from TEM images; the growth condition is the same as the initial 3 μm. (**d**) AFM image in AlN top surface. This image is the same sample as TEM images. The measurement areas of these AFM images are 1 μm^2^.

As shown in Fig. 4(b), there is a moving dislocation for the in-plane axis for the high-temperature growth at 1700 °C, and there is a possibility of a basic technique for the reduction of the dislocation. Therefore, the characteristic region of the TEM dark-field image shown in Fig. 5(a, b) with g/3, g = [0002] and g = [1–100], respectively. Because the *m*-axis dislocation length is micron level, and the TEM sample thickness at the depth axis is 300 nm, both types of the dislocations corresponding to burgers vectors visibly develop along the m-axis. According to the previous research, coupled grain by tiny grain at 3-dimensional growth in AlN growth is derived decrease of dislocation^31^; the dislocation behavior in high-temperature growth is regarded as characteristic in this study. Figure 5(c) indicates the atomic force microscopy (AFM) image in case of taking-off from the reactor after AlN growth at an initial 3 μm by high growth rate. The surface is atomically flat, and an atomic step as one monolayer of AlN is observed. There are two types of steps with a high amount of energy in step edge^30,34^, namely the zigzag and linear steps (Fig. 5c). Figure 5(d) shows an AFM image of the AlN surface after 10 μm growth, as shown in Fig. 3. The linearly diatomic layer step is confirmed by the growing zigzag type step. These findings in this study show that a growth temperature of 1700 °C with a clarified temperature of adatom desorption for AlN growth is unique in the reducing dislocations, surface morphology, and growth mode.

## Summary

In conclusion, a high-quality AlN layer is proven to develop on the c-plane sapphire at a high temperature of 1700 °C via MOVPE with the parasitic interactions between TMA and ammonia resolved. Additionally, the desorption of adatom is verified over 1550 °C, which is considered crucial for crystal growth on MOVPE growth in AlN. Furthermore, the low dislocation density in AlN at only a quarter-hour is realized with a dislocation density of less than 1 × 10^9^ cm^−2^. However, the dislocation grew along the m-axis at a low growth rate region of 1700 °C. Therefore, the dislocation density in increased AlN at 10 μm is obtained at approximately 4 × 10^8^ cm^−2^.

## Method

The MOVPE system reported in a previous study^23^ was used. The TMA and ammonia were used as aluminum and nitrogen precursors. Hydrogen is used to remove the influence V/III ratio by nitrogen decomposition. The TEM images were estimated by Material Science and Technology of Japan (MST). The atomic layer and surface roughness in AlN were measured via AFM, a product of HITACHI corporation 5500 M. An optical interference film thickness meter was used to measure the thickness of the AlN layer.

## Data Availability

The data that support the findings of this study are available from the corresponding author upon reasonable request.
